# Medicine

**Published:** 1912-09

**Authors:** J. A. Nixon


### Progress of the Medical Sciences.

### MEDICINE.

**Gastric Analysis.**—The value of analysis of test meals and stomach contents can only be appreciated properly provided we can compare the findings with a sufficiently large number of observations of normal and abnormal conditions to furnish a standard of averages. Panton and Tidy[1] have published some useful studies in gastric analyses, which may be tabulated as follows:—

1. *Normal Subjects.*
   Günzberg's Test +. Free HCl.=0.08 to 0.12.
   Total acidity 40 to 50.

2. *Carcinoma of Stomach.*
   Günzberg's Test nearly always −, *i.e.* Free HCl=0.
   Some dimethyl acidity often present.

3. *Simple Gastric Ulcer.*
   Günzberg's Test +. Free HCl. (average)=0.13.
   Total acidity 58.
   Free HCl. absent after severe hemorrhage.
   Free HCl. absent after previous gastro-jejunostomy.

4. *Duodenal Ulcer.*
   Günzberg's Test +. Free HCl. (average)=0.17.
   Total acidity 69.

5. *Free HCl. usually absent in carcinoma of the stomach, achylia gastrica, chronic alcoholic gastritis, severe anæmia, chronic pulmonary tuberculosis, and some forms of chronic dyspepsia.*

So far as averages can be of assistance, these figures will be found useful for reference. When, however, the limits of variation are considered, it will be seen that the argument from the general average to the particular case is likely to prove unreliable.

### Maximum and Minimum Findings.

1. *Carcinoma.*

| Free HCl. | Total Acidity. |
|---|---|
| Maximum=0.14. | Maximum=70. |
| Minimum=0. | Minimum=0. |
| (Absent in 39 cases out of 43.) | |

2. *Simple Gastric Ulcer.*

| Free HCl. | Total Acidity. |
|---|---|
| Maximum=0.21. | Maximum=86. |
| Minimum=0.06. | Minimum=34. |

3. *Duodenal Ulcer.*

| Free HCl. | Total Acidity. |
|---|---|
| Maximum=0.3. | Maximum=105. |
| Minimum=0.08. | Minimum=43. |
| (Total absence in 1 case.) | |

The *total acidity* figure is the number of c.c. of decinormal soda solution required to neutralise 100 c.c. of the given test meals.

The *free HCl.* is given in percentage terms, one litre of decinormal soda solution being required to neutralise 3.65 gm. of HCl.[2]

The *test meal* used by Panton and Tidy was practically that of Ewald, and consisted of two large cups of tea, with milk and sugar if desired, and two rounds of toast lightly buttered. The stomach was washed out a few hours before the test breakfast was given, and the gastric content was withdrawn by siphonage one hour afterwards without the addition of water. It was then filtered, and the filtrate and residue examined by various tests. The following simple analyses were always performed :—

1. With a portion of the filtrate Günzberg's phloroglucin vanillin test was performed in the usual manner, a positive reaction being taken as an indication of the presence of HCl.

2. To 10 c.c. of the filtrate, well diluted with water, three drops of dimethylamidoazobenzene were added, and decinormal soda run in from a burette until the pink colour was converted into yellow. This point coincides with the neutralisation of free HCl. in a solution of known strength. From the amount of decinormal soda used the amount of free acid was calculated, and when the Günzberg reaction was positive was taken to indicate HCl. The normal acidity by this method is the equivalent of 0.1 per cent. free HCl.

3. After neutralisation to dimethyl three drops of phenolphthalein were added to the same solution. Decinormal soda was run in until the first appearance of a permanent faint pink colour, the total acidity being given as the number of c.c. which neutralise 100 c.c. of the given test meal. The normal total acidity varies between 40 and 50.

**X-rays in gastric diagnosis** have been proved by Hertz[3] to be of the greatest value. Our knowledge of the anatomy and physiology of the stomach has been revolutionised as the result of this method of investigation.

The method of investigation consists in the administration of two ounces of bismuth oxychloride in bread and milk or porridge, and observing the shadow of the bismuth with X-rays either directly upon the fluorescent screen or on photographic plates. The patient should be examined both standing and lying down, and at varying intervals.

The *fundus*, which lies beneath the left dome of the diaphragm, and always contains gas, passes into the body of the stomach at the level of the cardiac orifice. In the vertical position the body is nearly of uniform width, is situated entirely to the left of the middle line, and is either vertical or inclines slightly to the right. It is separated from the *pyloric part* of the stomach by the *incisura angularis* on the lesser curvature (the pre-pyloric sphincter[4]). The pyloric part consists of the *pyloric vestibule* (or *antrum*), which is directed upwards as it passes to the right of the middle line and the *pyloric canal*, which is about one inch in length, passes backwards upwards, and to the right, ending in the duodenum, and is normally empty from contraction of the sphincter, even when the rest of the stomach is full. With the X-rays any abnormality in the position or shape of the stomach can be observed, but it must be borne in mind that the skiagram will show a material difference in the position of even a normal stomach, according to the vertical or horizontal attitude of the patient. Hour-glass contraction can be readily recognised, and a functional contraction of this nature may be distinguished from the organic form by the fact that in the latter the neck does not pass from the most dependent part of the upper segment.

Adhesions leading to fixation or deformity of some part of the stomach can be recognised with the X-rays, but they give no signs distinctive of cancer. Atonic and obstructive dilatation, with or without ptosis, are clearly displayed. Hypertonus, a sign commonly caused by duodenal ulcer, and rarely present in gastric ulcer, is easily diagnosed by the fact that the greater curvature does not reach the umbilicus in the vertical position.

The activity of peristalsis can be determined by X-rays, as well as the rate of evacuation of the stomach.

The **glycyl tryptophane test for the diagnosis of gastric carcinoma** has been again reported upon favourably by Neubauer and Fischer,[5] who conclude as follows: "Normal gastric juice does not split glycyl-tryptophane. The stomach contents in definite carcinoma gives almost regularly a positive reaction" (84 per cent. of cases verified by autopsy, 75 per cent of cases diagnosed clinically). "Other diseases of the stomach were as a rule negative."

*The tryptophane test*[6] depends on the power of ferments present in the cancer juices to break down proteins into amino acids. Tryptophane is an amino-acid which is split out of the protein molecule by trypsin and by the autolytic enzymes of tissue cells, whether they be normal or cancer cells; but the pepsin of normal gastric juice does not disintegrate proteins as far as the amino-acids, and hence tryptophane is not found in normal stomach contents. When disintegrating cancer tissue is present in the stomach, autolytic enzymes may be present in the gastric contents, and may set free tryptophane from the digesting protein. If it has not already been formed, it is necessary to add a compound of tryptophane, glycyl-tryptophane, to the filtered stomach contents and incubate for twenty-four hours before testing. During incubation the glycyl-tryptophane is split into glycin (amino-acetic acid) and tryptophane.

The test for tryptophane is made by adding bromine water cautiously, drop by drop, to 3 or 4 c.c. of the incubated stomach contents, to which a few drops of 3 per cent. acetic acid have been added. A reddish violet colour indicates the presence of tryptophane. The tryptophane reaction is not characteristic of cancer unless the possible presence of the proteolytic ferment of the pancreas or intestine is excluded.

Weinstein[7] claims to have shown that the use of glycyl tryptophane is superfluous for the detection of tryptophane-producing enzyme in gastric contents. Gastric contents always contain proteoses and peptones that are readily convertible by cancer enzyme into their corresponding simpler peptides and amino acids, including tryptophane.

Austin[8] considers the tryptophane test the most available one for cancer that can be used in a clinic.

It is doubtful how far this test depends on the presence of gastric carcinoma. Walker Hall and Scott Williamson[9] (and more recently Smithies[10]) have suggested that it may indicate nothing else than the presence of blood serum.

**Symptomatology of Gastric and Duodenal Ulcers.**—From a careful study of one thousand cases of ulcers of the stomach and duodenum Friedenwald[11] draws the following conclusions.

1. In patients suffering from various gastric disturbances 7.8 per cent. are affected with ulcers.

2. The largest proportion of ulcers occur between the twentieth and fiftieth year of age.

3. More than twice as many males are affected as females.

4. Anæmia is present in a large proportion of the cases of ulcers.

5. A history of over-indulgence in food or drink can be obtained in almost half of the number of cases of ulcer.

6. Acidity. The greatest proportion of cases of ulcer presents a normal acidity, *i.e.* 46 per cent., 30 per cent. a hyper-acidity, 23 per cent. a subacidity. Hyperacidity is proportionately more frequently observed in males and subacidity in females. In acute ulcers and those accompanied by recent hemorrhages the acidity is very high, while in chronic forms the acidity is low.

7. The average duration of symptoms is twelve years.

8. The most prominent symptom of ulcer, pain, occurs in 94 per cent. of cases, and pain is most frequent in cases associated with a high acidity. Pain appears sometimes immediately after food (gastric ulcer) or long after food (duodenal ulcer). Varying periods of intermission of pain as well as the other symptoms may occur.

9. An epigastric tender area is present in at least 90 per cent. of all cases, a dorsal tender area in 32 per cent.

10. Vomiting is a very prominent symptom occurring in 67 per cent. of cases.

11. Hæmatemesis is present in 22 per cent. of cases and melæna in 51 per cent. Melæna is more than twice as frequent as gastric hemorrhage. Occult blood is present in 81 per cent. of the cases.

12. Of the one thousand cases 52 per cent. are duodenal and 40 per cent. gastric, the largest proportion occurring in males (58 per cent.).

13. Acidity in duodenal ulcers: 48 per cent. present normal acidity, 35 per cent. hyperacidity, and 16 per cent. subacidity, hyperacidity being more frequently observed in males and subacidity in females.

14. Pain is present in 96.5 per cent. of duodenal ulcers, and is most prominent in cases with hyperacidity.

15. Distinct periods of intermission from pain and other symptoms, varying from one to twelve months or more, are exceedingly common in this affection.

16. Epigastric tenderness is present in 89 per cent. of the duodenal cases; a tender area to the right or left of the median line in 7 per cent.

17. Vomiting occurs in 21 per cent. of duodenal cases, and is more frequent in those accompanied by high acidity.

18. Melæna occurs in 54 per cent. of duodenal cases, and occult blood is present in 83 per cent.

**Sensibility of the Alimentary Canal.**—Hertz,[12] after investigating the sensibility of the alimentary canal to stimuli of various kinds, has arrived at the following interesting conclusions:—

1. The mucosa from the upper end of the œsophagus to the junction of the rectum with the anal canal: Tactile stimulus, negative.

2. Œsophagus and anus: Thermal stimulus, positive.
 Stomach and intestines: Thermal stimulus, negative.

3. Œsophagus and stomach: Dilute HCl. and organic acids, negative.
 Rectum: Glycerine, negative.
 Anus: Glycerine, positive.
 Whole mucosa: Alcohol, positive (sense of heat).

4. The surface of ulcers (gastric and intestinal) is no more sensitive to tactile, thermal, and chemical stimuli than the intact mucosa.

5. The sense of fulness is due to slow increase in tension exerted on the fibres of the muscular coat. There is an adequate tension which is constant for each segment. The volume of contents required to produce this tension varies with the tone of the muscle fibres.

6. The sense of fulness in the rectum has a special character by virtue of which it produces the call to defacation.

7. Hunger is a combination of the general sense of malaise and weakness with the local sense of an empty stomach. The latter sense is due to the periodical motor activity of stomach and intestines during fasting, in which a condition of muscular hypertonus and nervous hyperexcitability exists.

8. The only immediate cause of true visceral pain is tension exerted on the muscular coat of hollow organs and on the fibrous capsule of solid. In the alimentary canal pain is experienced when there is a more rapid or greater increase in the tension of the muscular fibres than that of the adequate tension for the sense of fulness (see above, No. 5).

9. Pain in diseases of the alimentary canal, although frequently true visceral pain, is sometimes due to the spread of disease to surrounding tissues or tension on peritoneal attachment, or pain of nervous origin referred to skin, muscles and connective tissue.

10. Tenderness generally depends on hyperalgesia of the skin, muscles and connective tissue supplied by the same segment of the central nervous system. External pressure may increase the tension referred to in No. 9.

11. All these various sensibilities may be exaggerated by training in neurasthenia.

J. A. Nixon.

REFERENCES.

1 Panton and Tidy, *Quart. J. Med.*, 1910–11, iv. 449.

2 Hutchison and Rainy, *Clinical Methods*, 5th Edition, p. 85.

3 Hertz, *Brit. M. J.*, 1912, i. 225.

4 Cathcart, *J. Physiol.*, 1911, xlii. 93.

5 Neubauer and Fischer, *München. med. Wchnschr.*, 1911, lviii. 674.

6 *J. Am. M. Ass.*, 1911, lvii. 1305.

7 Weinstein, *J. Am. M. Ass.*, 1911, lvii. 1420.

8 Austin, *Med. Rec.*, 1911, lxxx.

9 *Bristol M.-Chir. J.*, 1911, xxix. 96.

10 Smithies, *J. Am. M. Ass.*, 1912, lix. 539.

11 Friedenwald, *Am. J. M. Sc.*, 1912, cxliv. 157.

12 Hertz, Goulstonian Lectures, London, 1911.

Schmidt's *Jahrb.*, 1912, cccxv, 20; Kadner, *Epitome of New Work on the Physiology and Pathology of the Digestive Organs.*

